# A New Method for Estimating the Coverage of Mass Vaccination Campaigns Against Poliomyelitis From Surveillance Data

**DOI:** 10.1093/aje/kwv199

**Published:** 2015-11-14

**Authors:** K. M. O'Reilly, A. Cori, E. Durry, M. Z. Wadood, A. Bosan, R. B. Aylward, N. C. Grassly

**Keywords:** acute flaccid paralysis, Bayesian analysis, oral poliovirus vaccine, poliomyelitis, vaccination coverage

## Abstract

Mass vaccination campaigns with the oral poliovirus vaccine targeting children aged <5 years are a critical component of the global poliomyelitis eradication effort. Monitoring the coverage of these campaigns is essential to allow corrective action, but current approaches are limited by their cross-sectional nature, nonrandom sampling, reporting biases, and accessibility issues. We describe a new Bayesian framework using data augmentation and Markov chain Monte Carlo methods to estimate variation in vaccination coverage from children's vaccination histories investigated during surveillance for acute flaccid paralysis. We tested the method using simulated data with at least 200 cases and were able to detect undervaccinated groups if they exceeded 10% of all children and temporal changes in coverage of ±10% with greater than 90% sensitivity. Application of the method to data from Pakistan for 2010–2011 identified undervaccinated groups within the Balochistan/Federally Administered Tribal Areas and Khyber Pakhtunkhwa regions, as well as temporal changes in coverage. The sizes of these groups are consistent with the multiple challenges faced by the program in these regions as a result of conflict and insecurity. Application of this new method to routinely collected data can be a useful tool for identifying poorly performing areas and assisting in eradication efforts.

Immunization is a proven health intervention that directly protects vaccinated individuals and the remaining population through herd immunity. Vaccination may be delivered through routine immunization or supplementary immunization activities (SIAs), including mass immunization campaigns. SIAs are carried out where routine immunization is known to be poor, where the vaccination policy has recently changed and the immunization status of the target population needs to be updated, or as part of an accelerated disease control effort such as those made to eliminate or eradicate specific pathogens (1). Routine vaccination of children against poliomyelitis is supplemented with mass immunization campaigns using the oral poliovirus vaccine (OPV) in many countries as part of the Global Polio Eradication Initiative (http://www.polioeradication.org/).

The coverage of an SIA, defined as the proportion of the target population successfully vaccinated by the end of the campaign, is an important quantity to know but is difficult to directly estimate. A crude estimate of coverage often used is the number of vaccine doses distributed divided by the size of the target population. This administrative coverage is error-prone, as it assumes no wastage of vaccines, ignores individuals receiving multiple doses, and relies on accurate estimation of the size of the target population (2). A more recent method of analysis combines stock data, the size of target populations, and World Health Organization estimates of coverage to determine heterogeneity in coverage, but it still requires accurate estimates of the target population size (3). Cross-sectional health surveys collect information on children's immunization histories based on parental recall, but these are infrequent and have not included some of the key areas for the poliomyelitis eradication program. Postcampaign surveys are often carried out to assess coverage after mass immunization; children are finger-marked when vaccinated, and the proportion of finger-marked children is estimated several days later by an independent team surveying households and local gathering places (4). These surveys are not reliant upon knowledge of the target population size and are considered more accurate than stock estimates. However, postcampaign surveys are expensive, are prone to an upward bias of estimates (because the surveyors may use the same community map as the vaccinators), and in insecure areas may be impossible to carry out (since July 2012, postcampaign surveys have been cancelled in some areas of Pakistan due to security concerns (5)).

Poliomyelitis persists in Pakistan because of a variety of issues: managerial and operational problems, active conflict and inaccessibility, and failure to identify and access at-risk but hard-to-reach populations (6). These issues have resulted in populations with low vaccine coverage and in some cases a suggestion that some groups of children are consistently less likely to be vaccinated during campaigns compared with the general population (6). We refer to these groups of children as “persistently undervaccinated groups.” An undervaccinated group may include children whose accessibility by vaccination teams is limited or who are reachable but whose caregivers choose not to have them vaccinated.

Mass campaigns with OPV typically target several million children over a period of 3–5 days, and detailed records of activities are available for most countries at the second administrative (district) level. Additionally, acute flaccid paralysis (AFP) is investigated in more than 100,000 children globally every year; most cases of AFP (at least 95%) result from causes other than poliovirus infection (7). These children undergo a detailed clinical and epidemiologic investigation, which includes questions posed to their caregiver on the number of OPV doses received through routine immunization and SIAs (and results are usually reported separately). Previous estimates of vaccination coverage from these data (referred to as crude estimates) have simply divided the reported number of SIA doses received by children with AFP by the number of SIAs occurring during their lifetimes in their districts of residence (8). However, because these crude estimates are typically summarized for children within a relatively wide age range (e.g., <2 years of age), they represent an average over a period of a few years, diluting any rapid changes in the coverage of campaigns.

Here we describe a novel statistical method that permits estimation of vaccination campaign coverage over time from routinely collected AFP data and apply it to surveillance data from Pakistan.

## METHODS

### Data

The clinical symptoms of poliomyelitis are characterized by the rapid onset of weakness, typically but not exclusively in the lower limbs, with involuntary muscle paralysis but no loss of sensation, progressing eventually to spasticity over the longer term—collectively known as AFP. AFP is not exclusively caused by infection with poliovirus but may be caused by a wide range of conditions, such as Guillain-Barré syndrome, trauma, and infection with other enteroviruses (9).

Surveillance for poliomyelitis is based on reporting of AFP by health-care providers and detailed investigations (7). Two stool samples are collected within 14 days of the onset of paralysis and more than 24 hours apart and are tested for the presence of poliovirus. At the time of investigation, an interview with the parents/caregivers of the child is conducted where information on the reported number of OPV doses received through routine immunization and SIAs is sought. Cases in which 2 adequate stool samples are found to be negative for both wild-type and vaccine-related polioviruses are defined as nonpolio AFP.

Health-care-seeking by caregivers means that children with AFP are frequently captured by the surveillance network. At least 1 case of nonpolio AFP is expected annually in a population of 100,000 children aged <15 years (in high-risk areas, the target rate is 2 cases per 100,000 children aged <15 years (10)). In many areas with poliomyelitis cases, AFP reporting exceeds 1 per 100,000 children aged <15 years as a result of enhanced surveillance and a high incidence of enterovirus infections and other causes of AFP. This means that cases of AFP are typically reported from most parts of a country and that areas with conditions favoring poliovirus transmission are often oversampled (8, 11).

Nonpolio AFP data from children born between January 2008 and December 2011 with onset of paralysis between January 2010 and December 2011, a subset of data previously described (8), were used in this analysis. Children without sufficient information on age, date of birth, district of residence, or OPV vaccination history were excluded. For each case of nonpolio AFP, the number of OPV doses received through SIAs, as reported by the caregiver, was compared with the SIA calendar for the district of residence. Data from Pakistan were grouped into the following regions: 1) Balochistan and the Federally Administered Tribal Areas (FATA); 2) Khyber Pakhtunkhwa (KP); 3) Sindh; and 4) Punjab and Islamabad (observations from Gilgit-Baltistan, Azad Jammu, and Kashmir were not included in the analysis, owing to the small number of eligible cases (*n* = 20)). The incidence of reported AFP was compared with census-based population estimates to determine the geographic representativeness of these data (12, 13).

We applied methods typically used to assess campaign success and compared them with our new approach (10, 14). For each region, we report the proportions of children aged 6–23 months with nonpolio AFP who received no OPV doses and more than 3 doses (with 95% bootstrapped confidence intervals). Children in this age group are of special interest to the Global Polio Eradication Initiative because they constitute the majority of cases of poliomyelitis (8). Crude coverage estimates are derived by dividing the reported number of OPV doses by the number of SIA campaigns and are reported by region and year (8). This crude estimate accounts for the increase in the number of reported doses with age, because the denominator also increases with age and reflects an average of past campaign coverage over a 2-year time frame.

### New methods

**Figure 1.**
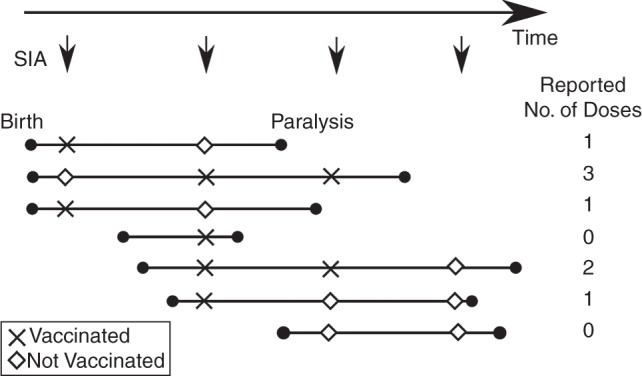
Schematic diagram of acute flaccid paralysis (AFP) data and the novel method the authors used for estimating oral poliovirus vaccine (OPV) immunization coverage in Pakistan. Lines representing the life histories of 7 children up to the onset of AFP are shown. The arrows at the top of the figure correspond to supplementary immunization activity (SIA), and the X's and diamonds for each child indicate whether the child was vaccinated during each SIA. Differences in the reported numbers of OPV doses received through SIA between children with AFP can be used to infer SIA coverage and is the basis for the new method described in the text (see Methods). In this example, 28.6% of children are reported to have received zero doses of OPV and no children are reported to have received more than 3 doses of OPV. In contrast to the new method, these summary statistics do not account for the age of each child, exposure to different numbers of SIAs, or errors in recall.

We report a new method for estimating vaccination campaign coverage which makes full use of the vaccination history of each child with AFP and the detailed SIA calendar (Figure 1). Campaign-specific estimates of vaccination coverage and identification of persistently undervaccinated groups are possible by making use of shared information among children with AFP who are exposed to common SIAs.

For example, child number 6 in Figure 1 was exposed to 3 SIAs and had 2 of those SIAs in common with child number 7. Differences in the number of reported doses provide information about the SIA that was not shared, while the average provides information about the SIAs the two children had in common. Because child 6 was reported to have received 1 dose of OPV and child 7 was reported to have received no doses, this suggests that the nonshared SIA had good coverage and the 2 shared SIAs had poor coverage.

To estimate vaccination campaign coverage from nonpolio AFP data, we developed a set of simple models of coverage that correspond to program experience, allowing for heterogeneity in space and time. We considered the effect of caregiver error in the recall and reporting of the number of OPV doses received by children with AFP by allowing the reported number of doses to differ from the actual number of doses received.

In our approach, each individual *i* present in the data is reported to have received *x_i_* doses of OPV, and from the vaccination calendar we calculate the expected number of SIAs (*s_i_*) from birth to the onset of paralysis. We assume that the reported number of doses is distributed around the actual number of doses (*y_i_*) according to a discretized lognormal distribution with a small coefficient of variation (α) to capture inaccuracies in the reported doses. We assume that the actual number of doses is binomially distributed, with the expected SIAs representing the number of trials and the probability of vaccination corresponding to campaign coverage. The proportion of children with AFP vaccinated during a campaign may vary between campaigns, even for campaigns with the same coverage, although this variation will be relatively small (approximately <5%) for typical estimates of coverage if the number of children included in the AFP sample is reasonably large (approximately >50). Full details on the methods are given in the Web Appendix (available at http://aje.oxfordjournals.org/).

Each model of vaccination coverage represents a different hypothesis about the cause of variability. The simplest *homogeneous* model assumes a constant probability (ρ) of being vaccinated during each campaign. To account for new initiatives or limitations in accessibility, the *homogeneous-temporal* model assumes a step change in vaccination coverage at a specific time point. Persistently undervaccinated groups are accounted for in the *heterogeneous* model, where the population is divided into 2 groups that differ in terms of their size and coverage. These groups may reside in the same geographic region but capture problems with inaccessibility or vaccine refusal. In this model, each child is arbitrarily assigned an additional variable describing his/her membership in either group (undervaccinated or not), and this variable is estimated using Markov chain Monte Carlo methods. The size of the undervaccinated group is estimated a posteriori from this augmented variable. The *heterogeneous-temporal* model accounts for both temporal changes in coverage and the presence of undervaccinated groups. Other models may be written as required to formally assess specific programmatic questions about changes in campaign coverage over time and/or location.

### Application to simulated and real AFP data

The parameters of each model consist of the coefficient of variation and the coverage parameters, summarized by θ = {ρ, α}. The posterior probability of the model, given the data, is

Pr(θ|X,Y,S)∝L(θ|X,Y,S)g(θ)=Pr(Y|θ)Pr(X,S|Y)g(θ),

where *L*(θ|*X*, *Y*, *S*) is the likelihood of observed and augmented data, given the model parameters, and *g*(θ) is the prior distribution of the model parameters. We calculated the posterior probability using the output from the Markov chain Monte Carlo methods, with each chain run for 2 million iterations with an initial burn-in of 500,000 iterations and thinned to every 100th iteration, leading to a final posterior sample of size 2,000 (15). Three chains were compared to ensure model convergence. The 4 models were compared using a rescaled deviance information criterion, which accounts for the fit of the model to the data and penalizes models with more parameters (16).

We used simulated data to assess the performance of our approach and the accuracy of model selection. We performed 100 simulations for each scenario (corresponding to the homogeneous, heterogeneous, homogeneous-temporal, and heterogeneous-temporal models, respectively, with varying parameter values), with a sample size of 200 children with AFP, broadly corresponding to the minimum annual sample sizes observed in each region of Pakistan. Each child was exposed to 15 sequential SIAs out of a potential 40 in the time series. These parameters were then estimated using our method, with model choice informed by the rescaled deviance information criterion.

We applied the method to AFP data from 2010–2011 for each region of Pakistan, and the fits of different vaccination models were compared using the rescaled deviance information criterion. Where there was evidence for a persistently undervaccinated group, estimates of the sizes of undervaccinated populations were explored for smaller geographic areas. For estimates of the number of children with AFP in the undervaccinated group, we included the additional uncertainty in the 95% credible intervals when extrapolating from the AFP sample to the general population by assuming that children with AFP represented a random sample from the general population.

The analysis was carried out with R statistical software, version 3.0.1 (R Foundation for Statistical Computing, Vienna, Austria). Institutional ethics approval was not sought, since this was a retrospective study. The databases we used are anonymized and free of personally identifiable information.

## RESULTS

### Summary of AFP data from Pakistan

**Figure 2.**
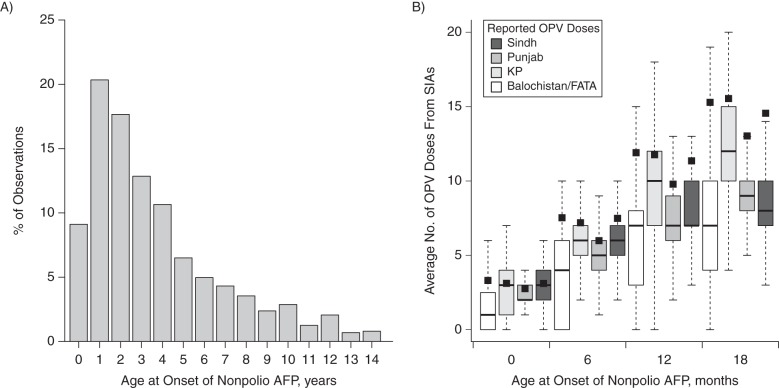
Age distribution of children with acute flaccid paralysis (AFP) not caused by poliomyelitis (nonpolio AFP) reported in Pakistan during 2010–2011 and their reported vaccination histories, by age. A) Age distribution; B) mean number of supplementary immunization activities (SIAs) experienced by children aged <2 years in different regions, by 6-month age group (black squares), together with the number of doses of oral poliovirus vaccine (OPV) they were reported to have received (boxes and whiskers). The boxes show the median value (black line) and interquartile range of the data, while the whiskers correspond to 1.5 times the interquartile range.

A total of 10,857 cases of AFP were reported in Pakistan between 2010 and 2011. Of these, 1,979 cases were not defined as nonpolio AFP, because they were classified as cases of poliomyelitis or cases compatible with poliomyelitis or because stool samples and information on the child's age were not available; these observations were excluded from the analysis. In the remaining 8,878 nonpolio AFP cases, the average rate was 7.2 cases per 100,000 population under 15 years of age, where 70.6% of cases were in children under 5 years of age (Figure 2A). Health authorities were notified of nonpolio AFP cases from across Pakistan, with a higher rate in Sindh and KP compared with other regions and a small amount of variation in the average age at onset of paralysis (Table 1). Of these cases, 2,753 children (31.0%) were born after January 1, 2008, and were under age 24 months at the time of AFP onset; details from these cases were used to infer vaccination coverage. The average number of SIAs children were exposed to increased with age and varied by region, reflecting differences in the vaccination calendar (Figure 2B). The number of reported OPV doses also increased with age, and the variability in dose reporting was larger in Balochistan/FATA and KP when compared with Sindh and Punjab.

**Table 1.** Surveillance Data on Cases of Acute Flaccid Paralysis (AFP) Not Caused by Poliomyelitis (Nonpolio AFP) With Onset of Paralysis Between 2010 and 2011 and Related Demographic Characteristics for Specified Regions of Pakistan

Region	Demography^a^		Surveillance Data^b^			
	Total Population (*N*)	No. of Children Aged <15 Years	No. of Nonpolio AFP Cases (2010–2011)	Annual Rate of Nonpolio AFP (per 100,000 Children Aged <15 Years)	Median Age of Nonpolio AFP Cases, months	No. of Children Aged <24 Months
Balochistan/FATA	12,325,103	4,363,086	612	7.0	39.4	273
KP	22,393,497	7,927,298	1,656	10.4	44.4	567
Sindh	38,537,646	13,642,327	2,315	8.5	49.3	1,324
Punjab	93,219,441	32,999,682	4,194	6.4	49.1	589
Other areas	7,117,313	2,519,529	101	2.0	48.1	—^c^
Total	173,593,000	61,451,922	8,878	7.2	47.7	2,753

Abbreviations: AFP, acute flaccid paralysis; FATA, Federally Administered Tribal Areas; KP, Khyber Pakhtunkhwa.

^a^ Total population based on the United Nations estimate for 2011 and regional breakdown provided by the most recent available census (1988).

^b^ Surveillance is regarded as sufficient when at least 2 cases of nonpolio AFP are detected per 100,000 population aged <15 years per year.

^c^ Observations from Gilgit-Baltistan, Azad Jammu, and Kashmir were not included in the analysis, owing to the small number of eligible cases (*n* = 20).

**Figure 3.**
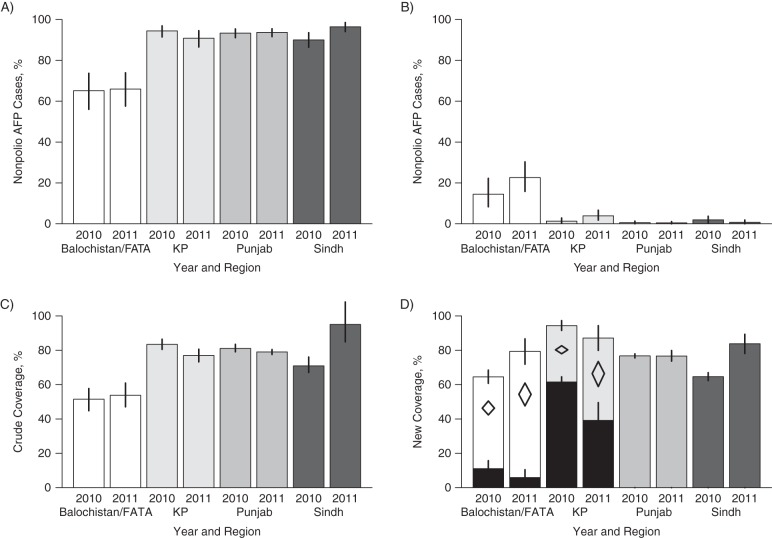
Summary statistics and estimates of oral poliovirus vaccine (OPV) immunization campaign coverage for specified regions of Pakistan, 2010–2011. The upper panels show the percentages of nonpolio acute flaccid paralysis (AFP) cases among children aged 6–23 months who were reported to have received more than 3 doses of OPV (A) and zero doses of OPV (B). Panel C presents crude estimates of vaccination coverage obtained by dividing the reported number of OPV doses by the number of immunization campaigns experienced by each child. Note that the upper limit can exceed 100%, reflecting error reporting. Panel D presents estimates of vaccination coverage obtained using the new method. In Balochistan/Federally Administered Tribal Areas (FATA) and Khyber Pakhtunkhwa (KP), the heterogeneous model gave the best fit, where 34% and 43% of children, respectively, were estimated to be in the undervaccinated group. Coverage in the undervaccinated group is shown by the black bars. The open diamonds indicate average coverage across the 2 groups in the heterogeneous model. The error bars in panels A–C indicate the 95% confidence intervals, and the error bars in panel D show the 95% credible intervals from the best-fit model.

The proportion of children aged 6–23 months in Balochistan/FATA who received more than 3 doses of OPV was 65% (95% confidence interval: 55, 74), while this estimate was higher in other regions (Figure 3A). Consistent with these results, the proportion of children who were reported to have received zero doses was higher in Balochistan/FATA (Figure 3B). Crude estimates suggested lower coverage in the Balochistan/FATA region (Figure 3C); in 2010, the crude estimate was 51.3% (95% confidence interval: 44.5, 57.8). Crude coverage estimates in KP were similar in value to the Punjab region, and there was an apparent increase in coverage in Sindh among children with nonpolio AFP in 2011 when compared with those who were paralyzed in 2010.

### Application of novel method to simulated data

**Figure 4.**
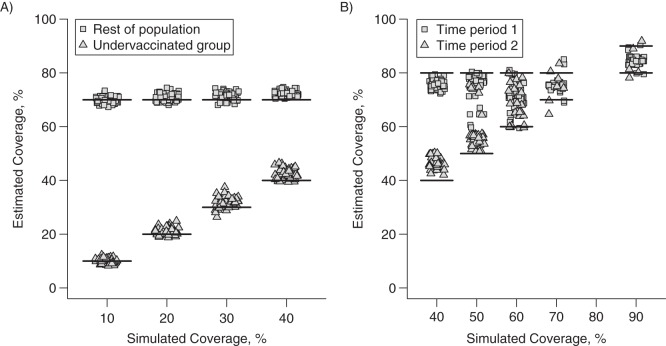
Model estimates for oral poliovirus vaccine coverage and actual simulated coverage. A) Data simulated under the heterogeneous model with 40% in the undervaccinated group; B) data simulated under the homogeneous-temporal model. Estimates are shown by the triangles and squares, and the actual value in each group or time period is shown by the horizontal lines. Results from 50 simulations are shown.

The novel method correctly identified the homogeneous model for data simulated under this model for a majority of simulations, and it provided accurate and unbiased estimates of coverage (see Table 2; full results are given in Web Table 1). The method was also able to detect persistently undervaccinated groups in at least 89% of simulations under the heterogeneous model for a variety of realistic parameter values (Table 2 and Figure 4A). Estimates of vaccination coverage in both the “covered” and undervaccinated groups were accurate and precise, and the sensitivity and specificity for detection of undervaccinated groups were above 90% in most simulations. Where the sensitivity or specificity was below 90%, the estimated coverage in the undervaccinated group was higher than simulated, and a larger proportion was estimated in the undervaccinated group.

**Table 2.** Performance of the Authors’ Novel Method When Applied to Acute Flaccid Paralysis Data Simulated Under a Homogeneous or Heterogeneous Model of Oral Poliovirus Vaccine Coverage

Vaccination Model and Coverage Parameter for Simulated Data				Best-Fit Model Based on rDIC (% of Simulations)^a^				Estimated Average Vaccination Coverage, %	
Campaign Coverage, %	Coverage in Undervaccinated Group, %	Proportion of Children in Undervaccinated Group, %	Average Coverage, %	Homogeneous	Heterogeneous^b^	Homogeneous-Temporal	Heterogeneous-Temporal^b^	Median	95%CrI
*Homogeneous Model*									
10			10	89	3	8	0	9.9	8.9, 11.0
50			50	27	57	16	0	50.2	48.3, 52.1
90			90	71	16	12	0	89.8	88.2, 91.4
*Heterogeneous Model*^b^									
70	10	10	64	0	100	0	0	63.6	60.4, 66.9
70	40	10	67	0	100	0	0	66.8	63.5, 70.1
70	10	40	46	0	100	0	0	46.3	43.5, 49.0
70	40	40	58	0	100	0	0	58.2	55.0, 61.4

Abbreviations: CrI, credible interval; rDIC, rescaled deviance information criterion.

^a^ Underlined values correspond to the correct model choice.

^b^ In the heterogeneous model, a proportion of the children with acute flaccid paralysis are assumed to come from an undervaccinated group (see Methods for details).

Temporal changes in coverage were also detected with good accuracy in the simulations (Table 3 and Figure 4B). Absolute changes in coverage that were greater than 10% were detected in at least 89% of simulations, and the 95% credible intervals of the estimated values generally included the simulated value. In simulations where undervaccinated groups were included in addition to temporal changes, model selection performed optimally when the absolute difference in coverage was greater than 20%. The estimates of coverage from the heterogeneous-temporal model fitted the simulated data well and maintained a high sensitivity and specificity for detecting undervaccinated groups. Where the simulated difference in temporal coverage was less than 20%, either the heterogeneous model or the homogeneous-temporal model was selected, suggesting that it was possible to detect undervaccinated groups or a change in coverage, but not both. Overall, coverage was underestimated, because the percentage of individuals in the undervaccinated group was overestimated.

**Table 3.** Performance of the Authors’ Novel Method When Applied to Acute Flaccid Paralysis Data Simulated Under a Homogeneous-Temporal or Heterogeneous-Temporal Model of Oral Poliovirus Vaccine Coverage^a^

Vaccination Model and Average Coverage Used in Simulations, %		Best-Fit Model Based on rDIC (% of Simulations)^b^				Estimated Average Vaccination Coverage, %			
Time Period 1 (12 Months)	Time Period 2 (12 Months)	Homogeneous	Heterogeneous	Homogeneous- Temporal	Heterogeneous- Temporal	Time Period 1		Time Period 2	
						Median	95%CrI	Median	95%CrI
*Homogeneous-Temporal Model*									
80	40	0	0	98	2	80.1	77.3, 82.9	40.4	37.3, 43.5
80	50	0	0	96	4	80.2	77.3, 83.1	50.3	47.1, 53.5
80	60	0	0	95	5	80.0	77.1, 82.9	60.1	56.8, 63.3
80	70	5	3	89	3	80.4	77.4, 83.2	70.1	66.8, 73.2
80	90	1	0	98	1	79.9	77.0, 82.6	89.9	87.1, 92.5
*Heterogeneous-Temporal Model*^c^									
52	28	0	0	0	100	45.1	41.9, 48.3	24.9	22.0, 27.8
52	34	0	0	0	100	44.9	42.1, 47.8	29.8	26.9, 32.8
52	40	0	0	0	100	44.5	41.8, 47.1	34.4	31.6, 37.2
52	46	0	37	31	32	44.5	42.0, 47.0	39.8	37.1, 42.5
52	58	0	13	35	52	44.3	41.8, 46.7	49.3	46.8, 51.7

Abbreviations: CrI, credible interval; rDIC, rescaled deviance information criterion.

^a^ These models assume that coverage varies between 2 time periods. In addition, in the heterogeneous-temporal model, a proportion of the children with acute flaccid paralysis are assumed to come from an undervaccinated group, as for Table 2 (see Methods for details).

^b^ Underlined values correspond to the correct model choice.

^c^ Assuming that 40% of children are in an undervaccinated group with 10% coverage that does not change over time.

### Applying the new methods to the Pakistan data set

The heterogeneous-temporal model had the best fit to the data from Balochistan/FATA and KP, while the homogeneous-temporal model had the best fit to Punjab and Sindh (Table 4). There was evidence in all regions of a change in SIA coverage in 2011 compared with previous years (2008–2010), and within Balochistan/FATA and KP there was evidence of a group that was persistently undervaccinated in comparison with the general population.

**Table 4.** Estimated Coverage of Oral Poliovirus Vaccine Immunization Campaigns in Pakistan Between 2008 and 2011 and the Size of the Undervaccinated Group in Specified Regions of Pakistan

Region and Year of SIAs	Total No. of Nonpolio AFP Cases	Estimated Vaccination Coverage^a^, %		Estimated Coverage in the Undervaccinated Group, %		Estimated Size of Undervaccinated Group, % of Population	
		Median	95% CrI	Median	95% CrI	Median	95% CrI
Balochistan/FATA							
2008–2010	273	64.5	60.8, 68.4	11	7.5, 15.7	34.2	28, 40.6
2011		79.4	71.9, 86.6	5.9	2.4, 10.4	34.2	28, 40.6
KP							
2008–2010	567	94.3	91.6, 97.3	61.6	58.2, 64.5	42.9	37.1, 48.8
2011		87.1	79.9, 94.3	39.2	26.4, 49.5	42.9	37.1, 48.8
Punjab							
2008–2010	1,304	76.7	75.6, 77.9				
2011		76.6	73.7, 79.9				
Sindh							
2008–2010	589	64.7	62.3, 67				
2011		83.8	78.2, 89.3				

Abbreviations: AFP, acute flaccid paralysis; CrI, credible interval; FATA, Federally Administered Tribal Areas; KP, Khyber Pakhtunkhwa; SIAs, supplementary immunization activities.

^a^ Excluding children in the undervaccinated group, where the best-fit model indicates heterogeneous coverage.

**Figure 5.**
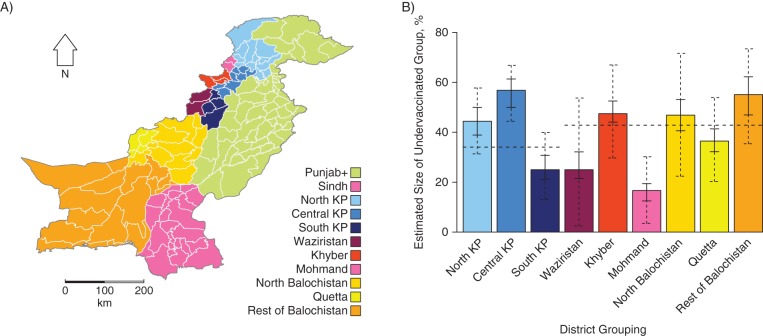
Sizes and locations of persistently undervaccinated groups of children under age 5 years in Pakistan, 2010–2011. Data on cases of acute flaccid paralysis (AFP) were aggregated for groups of districts (A). The estimated proportions of children persistently undervaccinated are shown by the colored bars (B). Regional averages for Khyber Pakhtunkhwa (KP; North, Central, and South), Balochistan (North, Quetta, and Rest of Balochistan), and the Federally Administered Tribal Areas (FATA; Waziristan, Kyber, and Mohmand) are shown by the dashed horizontal lines. The solid error bars indicate the 95% credible interval for the percentage of children in the AFP sample who were in the undervaccinated group, and the dashed error bars indicate the 95% credible interval for the population estimate, accounting for the fact that children with AFP were a random sample from a larger population.

Within Balochistan/FATA and KP, a majority of the target population (children aged <5 years) was estimated to have vaccination coverage above 50%, but 34% (95% credible interval (CrI): 28, 41) were undervaccinated in Balochistan/FATA and 43% (95% CrI: 37, 49) were undervaccinated in KP (Table 3, Figure 3D). When the 2 regions were divided into smaller areas, the Mohmand and Bajour districts (referred to as Mohmand) had a significantly lower proportion of undervaccinated children than the remaining districts within the Balochistan/FATA region (Figure 5). Central KP (consisting of 8 districts, including Peshawar and Kohat) was estimated to have an undervaccinated group that was significantly larger than those of other districts within the region, similar to that reported for the Balochistan/FATA region.

Within Punjab, coverage was estimated to be 76.7% (95% CrI: 75.6, 77.9) in 2010, with no change in the median value in 2011 but an increase in uncertainty and/or variability (median, 76.7%; 95% CrI: 73.7, 79.9). Within Sindh, coverage was 64.7% (95% CrI: 62.3, 67.0) in 2010, and it increased to 83.8% (95% CrI: 78.2, 89.3) in 2011. There was no evidence of an undervaccinated group within these regions.

## DISCUSSION

We have described a new method for estimating vaccination campaign coverage from routinely collected AFP data that allows the identification of persistently undervaccinated children as well as temporal changes in coverage while accounting for errors in reporting. Simulated data suggested that with limited reporting error, trends in vaccination coverage and the presence of undervaccinated groups can be accurately identified when the sample size exceeds 200. Heterogeneity in coverage from the presence of undervaccinated groups is epidemiologically important and impossible to assess using other methods. Application of these methods to Pakistan has illustrated that a large percentage of the Pakistan population is persistently undervaccinated and that coverage had changed in 2011 when compared with 2008–2010.

While current assessments of SIA performance (the proportions of children with more than 3 OPV doses, zero doses, and crude coverage estimates) are informative, they mask some of the heterogeneity present in the data and represent an average across children of different ages who have been exposed to different SIAs. It is clear from the crude analyses and our new model that Balochistan/FATA has suboptimal coverage and a persistently undervaccinated group of children, but the heterogeneity in coverage within KP was not apparent using the crude analyses, since the proportion of children with more than 3 doses was high and the proportion of zero-dose children was low. A high proportion of children (37%, 95% CrI: 37, 49) were estimated to be in a persistently undervaccinated group in central KP, with coverage of only 39.1% during SIAs in 2011. This highlights known issues with maintaining high vaccination coverage, such as in Peshawar, where there have been security concerns and where up to 20% of children were known to be inaccessible in 2009 (5). In Balochistan/FATA, inaccessibility is reported to be as high as 30% (5), which corresponds well to our estimates of the proportion in the persistently undervaccinated group (Table 4).

The existence of large undervaccinated groups in KP and Balochistan/FATA during 2008–2011 points to the multiple challenges the Global Polio Eradication Initiative has faced in Pakistan, including operational and managerial issues, lack of political and public support, natural disasters, inaccessibility, and conflict (17). While many of these challenges are being addressed, there have been serious concerns about the safety of the vaccination teams in some areas of Pakistan because of attacks that have occurred since 2012 (5). Furthermore, a ban on vaccination in North Waziristan has led to a major epidemic of poliomyelitis in FATA, and infection has spread to other parts of Pakistan and Afghanistan (18). Vaccination operations are reviewed by managers, and engagement with local communities and leaders occurs to help determine the reasons for poor coverage and work towards local solutions. Accurate estimates of vaccination coverage must form a key part of poliomyelitis eradication, and estimates will be updated beyond 2012 to support programmatic assessments in Pakistan and other countries.

Our method suffers from a number of limitations. The AFP dose reporting histories provide a relevant but convenient sample and may not fully represent all children who are targeted during immunization campaigns in Pakistan. The number of available AFP reports decreases as the spatial or temporal resolution is increased. This can be addressed by aggregating districts as in Figure 5 or by using spatial smoothing techniques that share information between nearby districts (e.g., conditional autoregressive modeling (19)). Large inaccuracies or bias in the reporting of vaccination histories from children with AFP will also prevent accurate estimation of SIA coverage using our method. We accounted for a degree of recall error, but we were unable to estimate the coefficient of variation for this distribution without a tightly constrained prior, reflecting confounding between variability in reporting and variability in coverage. We explored values for the coefficient of variation where the difference between the number of reported doses and the number of actual doses received was no more than 4, and within these limits the model was robust (Web Figure 1). In a previous study carried out in India, Grassly et al. (20) examined recall error by repeating interviews with parents and found that there was some recall error but no evidence of bias. It would be advantageous to estimate recall error in populations using these and similar methods, as this would improve the precision of model selection.

We were not able to explore variability in recall error between regions in addition to heterogeneity in vaccination coverage because of the difficulties involved in identifying children from undervaccinated groups when recall error is large. However, the identification of undervaccinated groups located in Balochistan/FATA and KP is consistent with the epidemiology of poliomyelitis in these regions, which continues to be reported in specific areas despite good campaign coverage on average across the region. Further studies, such as cross-sectional serological surveys, would be highly informative but difficult to implement because of the political instability in the area. Finally, a potential source of error in our estimates would be inaccuracies in the SIA calendar or long-term movement of children between regions, such that they were not exposed to the SIA reported for their district of residence. This would have been mitigated to some degree by the fact that SIA campaigns are often coordinated across regions.

Use of data augmentation techniques and Markov chain Monte Carlo methods has enabled us to develop a novel method for estimating vaccination coverage. This method may be a particularly useful programmatic tool for the late stages of the Global Polio Eradication Initiative, as it enables identification of poorly performing areas, taking advantage of data that are already routinely collected. Model validation using simulated data has illustrated the circumstances in which the analysis can correctly identify heterogeneity in coverage. Application of these models to surveillance data from Pakistan has highlighted considerable temporal and spatial variability in coverage between regions and the presence of persistently undervaccinated groups of children within certain key areas.

## Supplementary Material

Web Material
