# Supplementary material for: A New Method for Estimating the Coverage of Mass Vaccination Campaigns Against Poliomyelitis From Surveillance Data
Source: Am J Epidemiol. 2015 Nov 14;182(11):961–70. doi: 10.1093/aje/kwv199 (PMC4655745; doi:10.1093/aje/kwv199)
Supplement: Web Material [file supp_182_11_961__index.html]

A New Method for Estimating the Coverage of Mass Vaccination Campaigns Against Poliomyelitis From Surveillance Data — A New Method for Estimating the Coverage of Mass Vaccination Campaigns Against Poliomyelitis From Surveillance Data — Web Material 

# A New Method for Estimating the Coverage of Mass Vaccination Campaigns Against Poliomyelitis From Surveillance Data

## Web Material

Web Material

- Web Material - Pdf file
