# Supplementary material for: A New Method for Estimating the Coverage of Mass Vaccination Campaigns Against Poliomyelitis From Surveillance Data
Source: Am J Epidemiol. 2015 Nov 14;182(11):961–70. doi: 10.1093/aje/kwv199 (PMC4655745; doi:10.1093/aje/kwv199)
Supplement: Web Material [file supp_kwv199_kwv199supp.pdf]

Web Material for "A New Method for Estimating the Coverage of Mass Vaccination Campaigns Against Poliomyelitis From Surveillance Data"

K. M. O'Reilly, A. Cori, E. Durry, M. Z. Wadood, A. Bosan, R. B. Aylward, and N. C. Grassly

Correspondence to Dr. K. M. O'Reilly, Department of Infectious Disease Epidemiology, St. Mary's Campus, Imperial College London, Norfolk Place, London W2 1PG, United Kingdom (e-mail: k.oreilly@imperial.ac.uk).

WEB APPENDIX

Full description of the method

In this paper, we developed a statistical model to estimate vaccination coverage within specific time periods (here within the years 2008–2011). This differs from the crude estimates described in the main text of the manuscript which are an average of campaigns that children experience from birth to onset of paralysis. We assume that each child is exposed to a series of vaccination campaigns, and these campaigns may have different probabilities of "success," depending on the child's location, the year, and whether the child is classified as undervaccinated or not.

The data we use to estimate vaccination coverage are from surveillance for acute flaccid paralysis (AFP). As cases of nonpolio AFP are caused by a variety of infectious and noninfectious causes, we can use data from these cases to represent the vaccination histories of children in the general population. The average age of onset of nonpolio AFP is about 3 years, and it is the vaccination histories of these younger children that we want to use to estimate vaccination coverage of the general population. During AFP case investigation the caregiver of the affected child is asked to recall the number of doses of the oral polio vaccine (OPV) that the child has received. Vaccination cards are not always available; in the 2011–2012 DHS 36% of children surveyed had a vaccination card and there was considerable regional variation (2). A previous study in India documented recall error where there was no evidence of bias in reporting (3).

For each individual i ($i = 1,...,N$) where N is the total number of children with nonpolio AFP, let $z_i = (s_i, x_i)$ be the observed data, where each individual is exposed to s_i vaccination campaigns from the time they were born to the time of paralysis, and the caregiver of the child reports x_i doses of OPV received through SIAs. The function $p(x_i \mid y_i)$ describes the probability that the caregiver reports x_i doses given the true number y_i, which is unknown and considered as augmented data. We denote the vectors $Z = \{z_i; i = 1,...,N\}$ and $Y = \{y_i; i = 1,...,N\}$. We assume that reporting error follows a discrete log-normal distribution with median y_i and coefficient of variation α, which results in a distribution similar to the Poisson but with a variance different to the mean, and the variance is smaller than the mean if α is small in value (see Web Figure 1 for an example of the distribution of the reported doses when the true doses are 5 with a coefficient of variation of 0.1).

The variability in reported doses is summarized using the coefficient of variation of the corresponding continuous log-normal distribution. The distribution is defined as follows:

$$P(x_i \mid y_i, \sigma) = \int_{x_i - 0.5}^{x_i + 0.5} \frac{1}{x\sqrt{2\pi}\sigma} e^{\frac{-(\ln(x) - \ln(y_i))^2}{2\sigma^2}} dx,$$

which is defined by the median $ln(y_i)$ and standard deviation σ, which is then converted to the coefficient of variation $\alpha = \sqrt{e^{\sigma^2} - 1}$. We assume the median of this distribution is equal to the true number of doses received, in order to simplify the MCMC parameter estimation. This implies an upwards bias in reporting (ratio of median to mean = $e^{\sigma^2/2}$) although for the values of σ^2 that we estimate the extent of this bias is small (the difference between reported and true doses is no more than 4 when there are 10 doses are received). We report estimates of the coefficient of variation, where small values indicate low variation between observed and reported OPV doses.

Four statistical models were used to test hypotheses regarding heterogeneities in vaccination coverage. The first model assumes a *homogeneous* probability of being vaccinated; we assume the number of doses each child receives follows a binomial distribution with probability of vaccination ρ. All model parameters are contained within $\theta = \{\rho, \alpha\}$. A Bayesian framework was used, and consequently the posterior probability of the model parameters, given the data, is

$$\Pr(\theta \mid Z, Y) \propto L(\theta \mid Z, Y) g(\theta) \;,$$

where $L(\theta \mid Z, Y)$ is the likelihood of observed and augmented data given model parameters and $g(\theta)$ is the prior distribution of the parameters. For ρ, we assume an uninformative uniform prior on [0,1]. The priors for the standard deviation of the log-normal distribution for error reporting are denoted by μ_σ and σ_σ and were both log-normally distributed with a median of 0.1. In an extension of the *homogeneous* model, in the *heterogeneous model* "undervaccinated" group was considered in addition to the general population. Each child was assigned, as augmented data, an indicator variable w_i ($w_i \in \{0,1\}$) which indicates membership to a group (0 = "general population" and 1 = "undervaccinated") and coverage is given by

$$\tau(w_i) = \begin{cases} \rho, & \text{if } w_i = 0 \\ \nu, & \text{if } w_i = 1 \end{cases}$$

Consequently, for the heterogeneous model the parameters are defined as $\theta = \{\rho, \nu, \alpha\}$ and the augmented data comprise both *Y* and $W = \{w_i; i = 1, ..., N\}$.

Two additional models (*homogenous-temporal* and *heterogeneous-temporal*) were developed to further allow variation of vaccination coverage over time. In these models, we assumed a step function such that coverage was constant within predefined time periods. The augmented data included the true number of doses received within each time period, and the membership of a given group (either undervaccinated or not). Here we only considered two time periods but the model could be applied to more time periods if appropriate.

The likelihood for the homogeneous model (L_o) is given by

$$L_o(\theta \mid Z, Y) = \Pr(Y \mid \theta)\Pr(Z \mid Y, \theta)$$
$$= \prod_{i=1}^{N} \left(\begin{pmatrix} s_i \\ y_i \end{pmatrix} \rho^{y_i}(1-\rho)^{s_i - y_i} \int_{x_i - 0.5}^{x_i + 0.5} \frac{1}{x\sqrt{2\pi}\sigma} e^{-\frac{(\ln(x)-\ln(y_i))^2}{2\sigma^2}} dx \right)$$

In the remaining equations the discrete log-normal distribution is simplified to $\text{dlnorm}(x_i, y_i, \sigma)$. The likelihood of the homogeneous temporal (L_{ot}) model is as follows:

$$L_{ot}(\theta \mid Z, Y) = \prod_{i=1}^{N} \left(\begin{pmatrix} s_i^1 \\ y_i^1 \end{pmatrix} \rho_1^{y_i^1}(1-\rho_1)^{s_i^1 - y_i^1} \begin{pmatrix} s_i^2 \\ y_i^2 \end{pmatrix} \rho_2^{y_i^2}(1-\rho_2)^{s_i^2 - y_i^2} . \text{dlnorm}(x_i, y_i^1 + y_i^2, \sigma) \right),$$

where y_i^1 and y_i^2 are the number of doses received in the first and second series and s_i^1 and s_i^2 are the number of SIAs within the vaccine schedule, respectively.

The likelihood for the heterogeneous model (L_e) is

$$L_e(\theta \mid Z, Y, W) = \prod_{i=1}^{N} \left(\begin{pmatrix} s_i \\ y_i \end{pmatrix} (\tau(w_i))^{y_i}(1-\tau(w_i))^{s_i - y_i} . \text{dlnorm}(\text{x}_i, y_i, \sigma) \right).$$

The likelihood of the heterogeneous temporal model is given the data is as follows:

$$L_{et}(\theta \mid Z,Y,W) = \prod_{i=1}^{N} \left(\begin{pmatrix} s_i^1 \\ y_i^1 \end{pmatrix} \tau(w_i)^{y_i^1} (1-\tau(w_i))^{s_i^1 - y_i^1} \begin{pmatrix} s_i^2 \\ y_i^2 \end{pmatrix} \tau(w_i)^{y_i^2} (1-\tau(w_i))^{s_i^2 - y_i^2} .\text{dlnorm}(x_i, y_i^1 + y_i^2, \sigma) \right)$$

The parameters of the models were estimated using MCMC methods. A Metropolis-Hastings algorithm was used to update the augmented data and the standard deviation of error reporting, and a Gibbs sampler was used to update the coverage parameters (4). For the *homogeneous* and *homogeneous temporal* models the augmented data comprised only the true number of doses received: Y for the *homogeneous* model and $\{Y_1, Y_2\}$ for the *homogeneous temporal* model. For each iteration of the MCMC, an individual was randomly picked and a new value of y_i (denoted y_i*) was drawn by either increasing (with probability 0.5) or decreasing (with probability 0.5) the current value by one. The log-posterior probability was then recalculated and if higher than the current one, y_i* was accepted, and if lower, y_i* was accepted with a probability given by the ratio of the new and current posterior probabilities. As the update of y_i was not always symmetrical (due to upper and lower bound constraints on the possible number of doses received), a correction term was included in the acceptance ratio. For example, if y_i=0 and y_i*=1, then $\frac{Q(y_i \mid y_i^*)}{Q(y_i^* \mid y_i)} = \frac{0.5}{1} = 0.5$ (where Q denotes the proposal density distribution), and if y_i=14, y_i*=15 and s_i=15, then $\frac{Q(y_i \mid y_i^*)}{Q(y_i^* \mid y_i)} = \frac{1}{0.5} = 2$. For the *heterogeneous* and *heterogeneous temporal* model the augmented data further comprised of the categorisation of individuals as being in the general population or undervaccinated group, as denoted by w_i, and these augmented data were updated using the Metropolis-Hastings algorithm. As the move for w_i is always symmetrical (switching from 0 to 1 or *vice versa*) no correction term was required.

The coverage parameters were updated using a Gibbs sampler (4). Taking the posterior density of coverage in the homogeneous model as an example, using uniform priors on [0,1] for the coverage parameters, the marginal posterior distribution is:

$$P(\rho \mid Z,Y) \propto \Pr(Y \mid \rho) g(\rho)$$
$$= \prod_{i=1}^{N} \begin{pmatrix} s_i \\ y_i \end{pmatrix} \rho^{y_i} (1-\rho)^{s_i - y_i} \propto \rho^{\sum_{i=1}^{N} y_i} (1-\rho)^{\sum_{i=1}^{N} s_i - y_i} .$$

New values of coverage are therefore directly sampled from this beta marginal posterior distribution.

Traditionally, the deviance information criterion (DIC) is calculated as $DIC = \bar{D} + p_D$, where $\bar{D}$ is the expected deviance calculated over the posterior sample, and $p_D = \bar{D}\text{-}D(\hat{q})$, the effective number of parameters in the model, is estimated using the difference between the expected deviance $\bar{D}$ and the deviance of a certain parameter set $D(\hat{q})$, which can be chosen for instance as the mean or the mode of the posterior distribution (5,6). In models with no augmented data, the DIC is a robust method to assess competing models. However, DIC is known to be problematic for models with augmented data (6). In order to overcome this issue, we used a modified criterion defined as follows (and referred to as the rDIC):

- In a first step, the likelihood of all models with the exception of the homogeneous model were rescaled to be directly comparable to that of the homogeneous model. This was done by integrating over all augmented data not present in the homogeneous model. More specifically, the rescaled likelihoods for the four models were:

Heterogeneous model:

$$L_e(\theta \mid Z,Y,W) = \prod_{i=1}^{N} \left(\left(\begin{pmatrix} s_i \\ y_i \end{pmatrix} \rho^{y_i} (1-\rho)^{s_i-y_i} .(1-\sum w_i / N) + \begin{pmatrix} s_i \\ y_i \end{pmatrix} \nu^{y_i} (1-\nu)^{s_i-y_i} .\sum w_i / n \right) \text{dlnorm}(x_i, y_i, \sigma) \right)$$

Homogeneous temporal:

$$L_{ot}(\theta \mid X,S,Y) = \prod_{i=1}^{N} \left(\sum_{k=0}^{y_i} \left[\begin{pmatrix} s_i^1 \\ k \end{pmatrix} \rho_1^k (1-\rho_1)^{s_i^1-k} \begin{pmatrix} s_i^2 \\ y_i - k \end{pmatrix} \rho_2^{y_i-k} (1-\rho_2)^{s_i^2-y_i+k} \right] .\text{dlnorm}(x_i, y_i, \sigma) \right)$$

Note that if $y_i > s_i^1$, then $\begin{pmatrix} s_i^1 \\ k \end{pmatrix} r_1^k (1 - r_1)^{s_i^1 - k} = 0$.

Heterogeneous temporal model:

$$L_{et}(\theta \mid Z,Y,W) = \prod_{i=1}^{N} \left(\left(\sum_{k=0}^{y_i} \binom{s_i^1}{k} \rho_1^{\,k} (1-\rho_1)^{s_i^1-k} \binom{s_i^2}{y_i-k} \rho_2^{\,y_i-k} (1-\rho_2)^{s_i^2-y_i-k} (1-\sum w_i / \mathrm{N}) + \sum_{k=0}^{y_i} \binom{s_i^1}{k} \nu_1^{\,k} (1-\nu_1)^{s_i^1-k} \binom{s_i^2}{y_i-k} \nu_2^{\,y_i-k} (1-\nu_2)^{s_i^2-y_i-k} \sum w_i / N \right) . \sum_{i=1}^{N} \log(\mathrm{dlnorm}(x_i, y_i^1 + y_i^2, \sigma)) \right)$$

The mean deviance for each model, $\bar{D}$, was then calculated as -2 times the mean rescaled log-likelihood for that model, where the mean is taken over the posterior sample of parameters and augmented data.

- We evaluated the number of parameters for each rescaled model. All rescaled models have the same augmented data, but a different, well-defined number of parameters. We assumed that the augmented data would have the same contribution to the number of parameters in all models, so that, on a relative scale, the empirical number of parameters in each model would be 1 for the homogeneous model, 3 for the heterogeneous model, 2 for the homogeneous temporal model and 6 for the heterogeneous temporal model.
- Finally, the rDIC was calculated for each model as the sum of the rescaled mean deviance and the empirical number of parameters. For a given dataset, the model with lowest rDIC was selected, and if the difference in criterion between candidate models was less than 5, the most parsimonious model was selected.

We tested the ability of the rDIC to select the appropriate model on datasets simulated with a known model, and found that the rDIC was much more robust than the traditional DIC in the context of our method. We emphasize that further research on alternatives to DIC for models with augmented data should be carried out.

In simulations, error reporting was assumed to have a coefficient of variation (on the real scale) of 0.1. For each simulated dataset, when a model with an undervaccinated group was selected as the best fitting model, the posterior classification of individuals as undervaccinated or covered was compared to the simulations to assess the sensitivity and specificity of correctly identifying an undervaccinated individual.

Model sensitivity was defined as the probability that the correct model is selected given that the data were simulated under a specific model. Model precision (sometimes known as the

positive predictive value) is the probability that the correct model is selected given that all models are *a priori* equally possible.

Summary of the simulated data

- Homogeneous data: Homogeneous coverage where the proportion of children vaccinated at each SIA was 10%, 30%, 50%, 70% or 90% (5 scenarios).
- Heterogeneous data: Coverage in the general population was 70%. An undervaccinated group corresponding to 10, 20, 30 or 40% of children, with the proportion covered in this undervaccinated group equal to 0.1, 0.2, 0.3 or 0.4 (4x4 = 16 scenarios).
- Homogeneous-temporal data: Homogeneous coverage of 80% for the first 20 SIAs and of 40%, 50%, 60%, 70% or 90% for the next 20 SIAs (5 scenarios).
- Heterogeneous-temporal data: As the heterogeneous dataset but with an undervaccinated group, with coverage 10%, consisting of 0.40 of the population (5 scenarios).

The simulated data were used to test different scenarios as described in the manuscript. Many other scenarios have also been tested, for example to i) establish the minimum AFP sample size required to make satisfactory model inference (>200 cases) and ii) the effect of varying the error in the recall of the number of OPV doses by increasing the COV or using a Poisson distribution instead of the discretized log-normal. Use of a Poisson distribution (where the variance is equal to the mean) results in poor model inference. Increasing the COV when assuming a discrete log-normal results in increasingly poor model inference.

Web Table 1. Performance of the Authors' Method When Applied to AFP Data Simulated Under a Homogeneous or Heterogeneous Model of Vaccination Coverage

Vaccination Model and Coverage Parameter for Simulated Data				Best-Fit Model Based on rDIC (% of Simulations)[1]				Estimated Average Coverage (%)	
Campaign Coverage (%)	Coverage in Undervaccinated Group (%)	Proportion of Children in Undervaccinated Group (%)	Average Coverage (%)	Homo-geneous	Hetero-geneous	Homo-geneous–Temporal	Hetero-geneous–Temporal	Median	95% CrI
Simulations to test the homogeneous model									
**0.1**	-	-	0.1	89	3	8	0	0.099	0.089, 0.110
**0.3**	-	-	0.3	44	42	13	0	0.301	0.284, 0.318
**0.5**	-	-	0.5	27	57	16	0	0.502	0.483, 0.521
**0.7**	-	-	0.7	41	44	13	0	0.700	0.680, 0.718
**0.9**	-	-	0.9	71	16	12	0	0.898	0.882, 0.914
Simulations to test the heterogeneous model									
**0.7**	0.1	10	0.64	0	100	0	0	0.636	0.604, 0.669
**0.7**	0.2	10	0.65	0	89	11	0	0.650	0.617, 0.684
**0.7**	0.3	10	0.66	0	100	0	0	0.660	0.627, 0.694
**0.7**	0.4	10	0.67	0	100	0	0	0.668	0.635, 0.701
**0.7**	0.1	20	0.58	0	100	0	0	0.579	0.549, 0.611
**0.7**	0.2	20	0.6	0	100	0	0	0.601	0.569, 0.633
**0.7**	0.3	20	0.62	0	95	5	0	0.622	0.589, 0.654
**0.7**	0.4	20	0.64	0	100	0	0	0.639	0.605, 0.671
**0.7**	0.1	30	0.52	0	95	5	0	0.523	0.493, 0.552
**0.7**	0.2	30	0.55	0	100	0	0	0.554	0.524, 0.585
**0.7**	0.3	30	0.58	0	95	5	0	0.582	0.551, 0.614
**0.7**	0.4	30	0.61	0	100	0	0	0.610	0.578, 0.643
**0.7**	0.1	40	0.46	0	100	0	0	0.463	0.435, 0.490
**0.7**	0.2	40	0.5	0	100	0	0	0.502	0.473, 0.532
**0.7**	0.3	40	0.54	0	100	0	0	0.542	0.512, 0.573
**0.7**	0.4	40	0.58	0	100	0	0	0.582	0.550, 0.614

Abbreviations: AFP, acute flaccid paralysis; CrI, credible interval; rDIC, rescaled deviance information criterion.

In the heterogeneous model, a proportion of the children with AFP are assumed to come from an undervaccinated group (see Methods for details). A summary of these results is presented in the text (Table 2).

[1] Underlined values indicate the model from which the data set were simulated, and consequently should provide the best fit to the data.

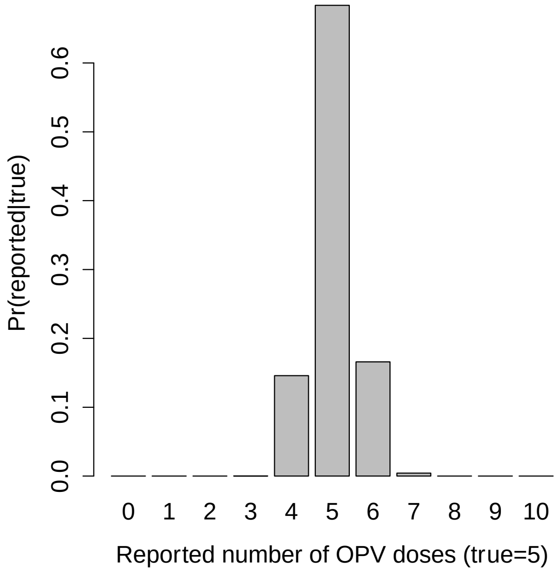

Web Figure 1. Example of a discrete log-normal distribution used for modeling the reported number of doses of oral poliovirus vaccine (OPV). The example is that of a child who received 5 doses of OPV; if the coefficient of variation were 0.1, the reported number of doses would vary from 3 to 7, accounting for some parents overestimating and some underestimating. More than 95% of the observations will lie between 4 and 6 reported doses of OPV.

References

1. Farag NH, Alexander J, Hadler S, Quddus A, Durry E, Wadood MZ, et al. Progress toward poliomyelitis eradication — Afghanistan and Pakistan, January 2013-August 2014. *MMWR Morb Mortal Wkly Rep* [Internet]. [cited 2014 Nov 6] 2014 Oct 31;63(43):973–977. Available from: http://www.ncbi.nlm.nih.gov/pubmed/25356605.

2. National Institute of Population Studies. *Pakistan Demographic and Health Survey 2012–13* [Internet]. Calverton, MD: ICF International; 2013 [cited 2014 Mar 24]. Available from: http://dhsprogram.com/pubs/pdf/FR290/FR290.pdf.

3. Grassly NC, Wenger J, Durrani S, Bahl S, Deshpande JM, Sutter RW, et al. Protective efficacy of a monovalent oral type 1 poliovirus vaccine: a case-control study. *Lancet* [Internet]. [cited 2013 Nov 13] 2007 Apr 21;369(9570):1356–1362. Available from: http://www.ncbi.nlm.nih.gov/pubmed/17448821.

4. Robert C, Casella G. *Introducing Monte Carlo Methods with R* [Internet]. 1st edition. New York, NY: Springer New York; 2010 [cited 2013 Nov 10]. Available from: http://link.springer.com/10.1007/978-1-4419-1576-4.

5. Spiegelhalter DJ, Best NG, Carlin BP, van der Linde A. Bayesian measures of model complexity and fit. *J R Stat Soc Ser B Stat Methodol*. 2002 Oct;64(4):583–639.

6. Celeux G, Forbes F, Robert CP, Titterington DM. Deviance information criteria for missing data models. *Bayesian Anal* [Internet]. [cited 2013 Dec 17] 2006 Dec 1;1(4):651–673. Available from: http://projecteuclid.org/euclid.ba/1340370933.
